# Effects of long-term antipsychotic medication on brain instability in first-episode schizophrenia patients: a resting-state fMRI study

**DOI:** 10.3389/fphar.2024.1387123

**Published:** 2024-05-23

**Authors:** Maoxing Zhong, Zhening Liu, Feiwen Wang, Jun Yang, Eric Chen, Edwin Lee, Guowei Wu, Jie Yang

**Affiliations:** ^1^ Department of Psychiatry, National Clinical Research Center for Mental Disorders, and National Center for Mental Disorders, The Second Xiangya Hospital of Central South University, Changsha, Hunan, China; ^2^ Department of Psychiatry, The University of Hong Kong, Pok Fu Lam, Hong Kong SAR, China

**Keywords:** antipsychotic drugs, negative symptoms, caudate, supramarginal gyrus, parahippocampal gyrus, insula

## Abstract

Early initiation of antipsychotic treatment plays a crucial role in the management of first-episode schizophrenia (FES) patients, significantly improving their prognosis. However, limited attention has been given to the long-term effects of antipsychotic drug therapy on FES patients. In this research, we examined the changes in abnormal brain regions among FES patients undergoing long-term treatment using a dynamic perspective. A total of 98 participants were included in the data analysis, comprising 48 FES patients, 50 healthy controls, 22 patients completed a follow-up period of more than 6 months with qualified data. We processed resting-state fMRI data to calculate coefficient of variation of fractional amplitude of low-frequency fluctuations (CVfALFF), which reflects the brain regional activity stability. Data analysis was performed at baseline and after long-term treatment. We observed that compared with HCs, patients at baseline showed an elevated CVfALFF in the supramarginal gyrus (SMG), parahippocampal gyrus (PHG), caudate, orbital part of inferior frontal gyrus (IOG), insula, and inferior frontal gyrus (IFG). After long-term treatment, the instability in SMG, PHG, caudate, IOG, insula and inferior IFG have ameliorated. Additionally, there was a positive correlation between the decrease in dfALFF in the SMG and the reduction in the SANS total score following long-term treatment. In conclusion, FES patients exhibit unstable regional activity in widespread brain regions at baseline, which can be ameliorated with long-term treatment. Moreover, the extent of amelioration in SMG instability is associated with the amelioration of negative symptoms.

## Introduction

Schizophrenia is a chronic and debilitating mental disorder associated with high recurrence rates, which leads to a progressive decline in patients’ functioning (Kissling, 1991; Dixon et al., 1999; Van Os and Kapur, 2009). First-episode schizophrenia (FES) refers to patients with an illness duration of less than 1.5 years (llison-Wright et al., 2008), typically observed during adolescence (Mackrell and Lavender et al., 2004). Early treatment can lay a strong foundation for patients’ prognosis, even in the presence of persistent symptoms (Tarcijonas and Sarpal, 2019). The primary method of treatment for schizophrenia involves antipsychotic drugs, which primarily target the dopamine D2 receptor in the brain (Kapur and Mamo, 2003), assisting in the restoration of abnormal brain areas and regulation of neural network function among FES patients.

Numerous studies have demonstrated the regulatory effects of antipsychotic drug therapy on brain function in patients. For instance, drug therapy has been found to modulate the connectivity of the default mode network (DMN) in individuals with schizophrenia (Sambataro et al., 2010; Guo et al., 2017; Guo et al., 2018). Additionally, evidence supports the improvement of connectivity in key brain regions including the striatum, hippocampus, and anterior cingulate cortex (Anticevic et al., 2015; Sarpal et al., 2015; Kraguljac et al., 2016b). Antipsychotic drugs not only regulate large-scale functional network abnormalities to some extent but also enhance the overall efficiency of the patient’s brain (Hadley et al., 2016; Crossley et al., 2017). Overall, these findings highlight the broad impact of antipsychotic drug therapy on brain function and its potential to improve connectivity in patients with schizophrenia.

However, the majority of studies in this field adopt a static perspective when investigating the impact of drugs on abnormal brain regions. It is important to note that the brain’s activity is dynamic, constantly changing over time (Calhoun et al., 2014; Liao et al., 2019). Consequently, time-averaged or static research approaches offer limited insights. Prior investigations have also examined the abnormal dynamic patterns of the brain in various mental disorders, including schizophrenia (Duan et al., 2020b) and major depressive disorder (Yang et al., 2022). As our understanding of brain dynamics deepens, we can gain a clearer understanding of the differences in brain activity among individuals with mental disorders. Hence, it is imperative to employ a dynamic approach in examining the potential impact of antipsychotic medication on brain stability in order to address the existing knowledge gaps in the relevant research domain.

Notably, most current research primarily focuses on the efficacy of short-term antipsychotic drugs in addressing brain abnormalities (Zeng et al., 2016; Zong et al., 2019). According to the available literatures, only five studies have investigated the long-term effects of antipsychotics on abnormal brain function in schizophrenia (Li et al., 2016; Guo et al., 2017; Li et al., 2018; Deng et al., 2022). Clinical guidelines around the world commonly recommend antipsychotic treatment for FES patients for a minimum of 6–24 months to achieve the desired therapeutic outcomes (Lehman et al., 2004; Buchanan et al., 2010; Galletly et al., 2016; Crockford and Addington, 2017). Therefore, it is of paramount importance to investigate the effects of long-term antipsychotic drugs (over 6 months) on brain structure and function. Exploring the effectiveness of long-term antipsychotic drugs on individuals with brain abnormalities holds great value in demonstrating the significance of maintenance therapy and providing novel insights for drug development.

This study aims to investigate the effects of long-term antipsychotic use on the brain regional activity stability of FES patients. The fractional amplitude of low-frequency fluctuations (fALFF) is a measure that focuses on regional fluctuations in brain activity based on blood oxygen level dependent signals. The dynamic fALFF (dfALFF) delineates the time-varying brain regional activity maps across sliding-windows. The coefficient variation of dfALFF (CVfALFF) reflects the brain regional activity stability across sliding-windows. Here, we adopted the CVfALFF to examine the effect of long-term antipsychotic therapy on the brain regional activity stability of FES patients. Following established clinical guidelines, we treated 48 FES patients for a duration of 6–24 months. Our study had three specific aims: 1) To identify areas showing abnormal stability of regional activity at baseline in FES and investigate their relationship with patients’ clinical symptoms, 2) To explore the long-term effects of antipsychotics on detected areas showing abnormal stability of regional activity in patients, and 3) To examine the correlation between the changes of regional activity stability in detected brain regions and the recovery of clinical symptoms.

## Materials and methods

### Participants

There were 105 participants (52 patients with FES) from three datasets: The Second Xiangya Hospital (Changsha, Hunan Province, China, Dataset #1 and Dataset#2), and Queen Mary Hospital, The University of Hong Kong (Hong Kong, China, Dataset #3). Information for each dataset is presented in Supplementary Table S1, and criteria for patient acceptance and exclusion are presented in Supplementary Methods S1. The Structured Clinical Interview of the DSM-IV-patient version (SCID-P) was used to recruit patients with FES. According to the previous studies (Anticevic et al., 2015b; Zhang et al., 2021), the sources of different datasets were included as covariates in further fMRI imaging data analysis. The severity of the patient’s clinical symptoms is assessed using the Scale for the Assessment of Positive Symptoms (SAPS) (Andreasen et al., 1995) and the Scale for the Assessment of Negative Symptoms (SANS) (Andreasen, 1989) by highly trained psychiatrists. All participants completed clinical symptom and resting-state fMRI measurements at baseline, with 24 patients completing follow-up surveys after continuing treatment for 6 months.

Researchers track medication status every 2 months through face-to-face interviews or phone calls. Conduct face-to-face interviews with patients who visit the hospital on time every 2 months for follow-up. In addition, for patients undergoing phone monitoring, we also attempt to contact the family members or guardians of the patients monitoring their medication treatment. In order to ensure that patients comply with treatment, we focused on asking some questions during medication monitoring process, such as daily drug dosage, treatment effectiveness, and side effects of the drug. To mitigate the effects of adjunctive therapies on patients’ brain function, we excluded individuals who received electroconvulsive therapy (ECT) and transcranial magnetic stimulation (TMS) treatments during the follow-up period. Finally, a total of 28 patients withdrew during follow-up. No significant difference in clinical symptom scores at the baseline was found between the dropped-out patients and follow-up patients (Supplementary Table S2). In addition, all patients are using second-generation antipsychotic drugs, and the dosage of the drugs is determined by a professional psychiatrist (Supplementary Methods S2). Conversion of drug dosage to chlorpromazine equivalence for each patient (50–1,000 mg/day).

We recruited 53 age- and gender-matched healthy controls from the local community using the SCID non-patient version for screening, none of whom had a first-degree relative with psychiatric disorders. We collected resting fMRI data from the healthy controls that met the standards. All participants have signed a voluntary informed consent form. The local ethics committee at each data collection point reviewed and approved the procedures and consent form.

### Image data acquisition and preprocessing

In this study, the resting-fMRI data of all participants were collected. During this process, participants need to maintain a flat and stable body, be alert, keep their eyes and mouth tightly closed, and try not to think of unnecessary thoughts. Soft earplugs and foam pads were used to decrease scanner noise and head motion. The parameter settings for both baseline and follow-up scans of each dataset are the same. Subsequently, we used Statistical Parametric Mapping 12 (SPM12, http://www.fil.ion.ucl.ac.uk/spm) and Data Processing Assistant for Resting-State fMRI (DPABI, http://www.rfmri.org/) (Yan et al., 2016) for image preprocessing. Detailed information on imaging parameters and preprocessing procedures for each clinical center is listed in the Supplementary Methods S3.

The exclusion criteria for sample selection included: 1) Head motions larger than a 2.5-mm translation or 2.5° rotation in any direction. 2) The fMRI data failed to normalize to MNI space which is visually inspected by an experienced data analyst. After quality control, a total of 22 patients at follow-up, 48 patients at the baseline and 50 HCs were included in the final analysis. Furthermore, head movement scrubbing regression was used to eliminate the confounding effect of subtle head movements (see Supplementary Methods S3). After pre-processing, the images were entered into the dfALFF calculation process.

### DfALFF calculation

We used the sliding window method in the Dynamic Brain Connectome (DynamicBC) toolbox to calculate dfALFF (Liao et al., 2014). In an optimal scenario, the window should be sufficiently large to enable a reliable estimation of fALFF and to distinguish the lowest frequencies of interest in the signal, while also being small enough to detect possible transient events of interest (Leonardi and Van De Ville, 2015; Li et al., 2019). In order to mitigate the risk of spurious fluctuations stemming from window lengths shorter than the fmin, our window length needs to exceed the threshold of 1/fmin, and fmin is the minimum frequency of time series (Leonardi and Van De Ville, 2015). In this study, our fmin is 0.01 Hz, so we determined a window length of 50 TRs (i.e., 100 s). In dataset #1, the time series consisted of 240 TRs (480 s), and the window was shifted by 1 TR (2 s). The full-length time series was then divided into 191 windows for each subject. In dataset #2, the time series consisted of 206 TRs (412 s), and the window was shifted by 1 TR (2 s). The full-length time series was then divided into 157 windows for each subject. In dataset #3, the time series consisted of 240 TRs (480 s), and the window was shifted by 1 TR (2 s). The full-length time series was then divided into 191 windows for each subject. Finally, we obtained the fALFF map corresponding to each sliding window and then calculated the coefficient of variation of fALFF (CVfALFF) maps to capture the stability of the brain regional activity. The calculation formula for CVfALFF is as follows:

$$CVfALFF=\frac{\sqrt{\left[(\sum \right.\left.\left(x\;-\;\mu \right)²)\;/\;N\right]}}{\left(\sum x\right)\;/\;N}$$

*x* represents the fALFF map for each sliding window, *μ* is the average fALFF value across sliding windows, and *N* is the total number of sliding windows.

**FIGURE 1:**
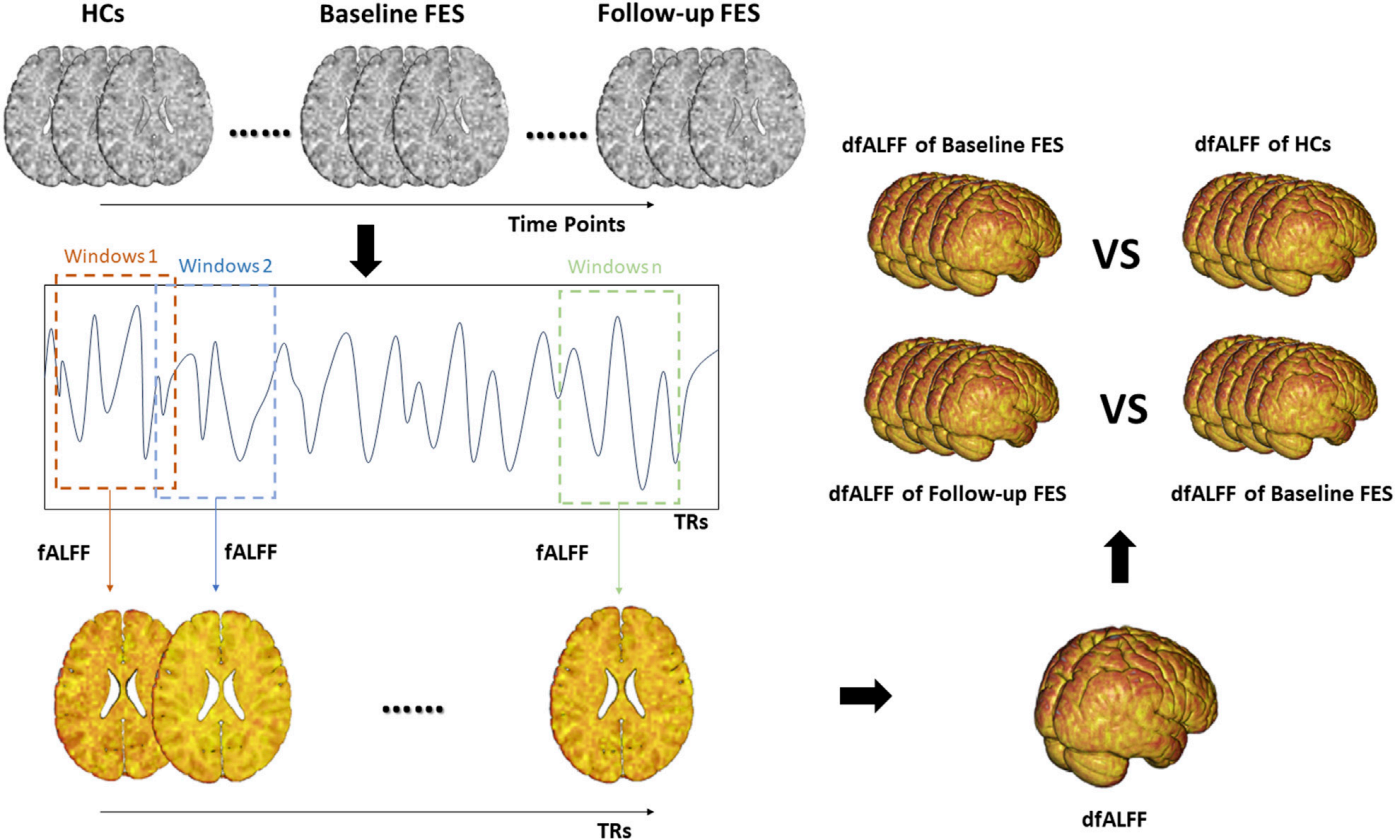
The calculation steps of dfALFF. Note. fALFF, fractional amplitude of low frequency fluctuations; dfALFF, dynamic fractional Amplitude of Low Frequency Fluctuations; FES, first-episode schizophrenia; TRs, repetition time.

The calculation steps are shown in Figure 1.

### Statistical analysis

We used SPSS 19.0 to analyze the demographic and clinical characteristics of the baseline group and HCs. We used the two-sample t-test for continuous variable analysis, and the Chi-square analysis for categorical variable analysis. The statistical threshold was set at *p* < 0.05.

Subsequently, we analyzed the fMRI data using SPM12 software. To identify areas of the brain where CVfALFF differed between HCs and patients, we used a two-sample t-test to analyze baseline patients (*n* = 48) and HCs (*n* = 50) with age, gender, education, and dataset location as covariates. The paired t-test was then performed within baseline and follow-up patients (*n* = 22), using the brain regions that differed from HCs at baseline as mask, to determine longitudinal changes in follow-up patients after treatment. After adjusting for false discovery rate (FDR) at voxel levels, the significance level of the above comparisons was *p* < 0.05. Of note, we also used Pearson’s correlation analysis to explore the correlation between changes in CVfALFF and changes in clinical symptoms in patients.

### Validation analysis

To verify the authenticity of the results, we reprocessed our data adopting a window length of 30 TRs to obtain the dfALFF again.

## Results

### Demographic and clinical characteristics

Table 1 shows the demographic and clinical characteristics of the data, indicating that there were no significant differences in age, sex, and education between patients and HCs at baseline. The mean duration of disease of the 48 FES patients was (4.46 ± 3.80) months, including (4.09 ± 4.13) months without treatment, and the total SAPS score at baseline was (26.79 ± 14.79) and total SANS score was (22.35 ± 16.20). It can be seen that after treatment, the total scores of SAPS and SANS in patients significantly decreased. The average reduction in SAPS total score was (18.81 ± 15.91), and the average reduction in SANS total score was (10.95 ± 14.05) (both *p* < 0.001). The average follow-up interval for patients is (11.49 ± 3.82) months. What’s more, a total of 20 patients experienced a decline of more than 30% in SAPS total scores and 17 patients experienced a decline of more than 30% in SANS total scores after treatment (Supplementary Table S3).

**TABLE 1:** Demographic data and clinical data of participants.

Characteristic	FES at the baseline *N* = 48	Healthy controls *N* = 50	Statistics (t/χ^2^)
Age (years)	23.33 ± 6.27	24.48 ± 5.67	−0.95
Sex (male: female)	17:21	23:27	1.06
Education (years)	12.79 ± 3.10	13.34 ± 2.02	−1.04
During of illness (months)	4.46 ± 3.80	-	
duration of untreated psychosis (DUP)	4.09 ± 4.13	-	
Clinical symptom scores			
SAPS total scores	26.79 ± 14.79	-	
SANS total scores	22.35 ± 16.20	-	

Characteristic	FES at follow-up *N* = 22		statistics (t/χ^2^)
	T1	T2	
CPZ equivalents (mg)	—	210.35 ± 142.54	
Average follow-up interval (months)		11.49 ± 3.82	
Clinical symptom scores			
SAPS total scores	23.19 ± 14.42	4.75 ± 8.03	^a^5.29***
SANS total scores	22.89 ± 16.43	11.95 ± 10.17	^a^3.40**

FES, first-episode schizophrenia patients; T1, time point at the baseline; T2, time point after treatment; DUP, duration of untreated psychosis; CPZ, chlorpromazine; SAPS, scale for the assessment of positive symptoms; SANS, scale for the assessment of negative symptoms.

^a^ Paired t-test in FES, patients between the baseline and follow-up.

***p < 0.001; **p < 0.01.

### Abnormal dfALFF in the FES patients at the baseline

**FIGURE 2:**
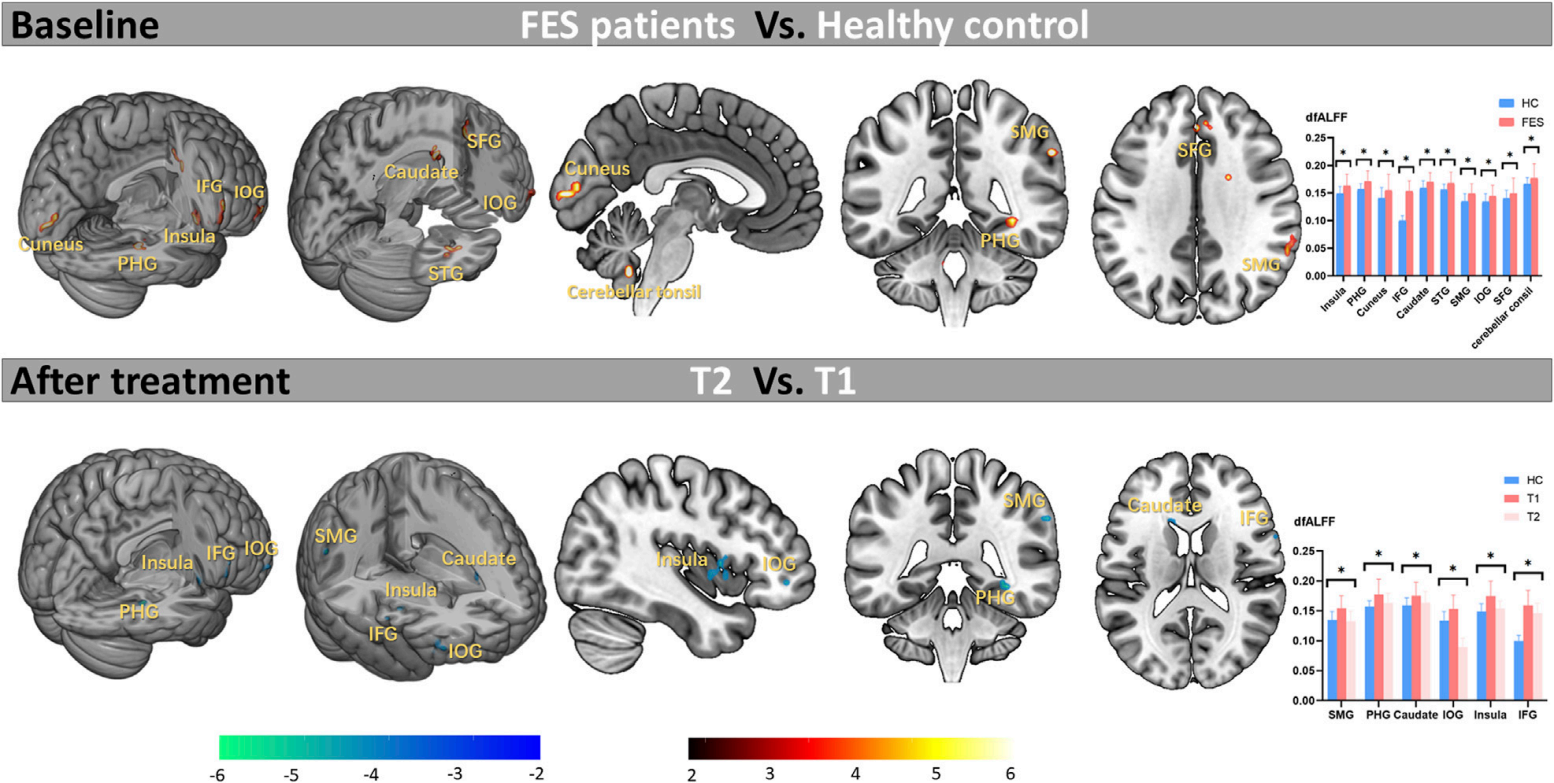
Abnormal variation of activity at baseline and effects of long-term antipsychotic drugs on abnormal brain areas in FES patients. Note. FES, first-episode schizophrenia; HC, healthy control; T1, time point at the baseline; T2, time point after treatment; dfALFF, dynamic fractional Amplitude of Low Frequency Fluctuations; *p < 0.05 (corrected by FDR); PHG, parahippocampal gyrus; IFG, inferior frontal gyrus; STG, superior temporal gyrus; SMG, supramarginal gyrus; IOG, orbital part of inferior frontal gyrus; SFG, superior frontal gyrus.

At the baseline, compared with HCs, FEP patients showed significant increased CVfALFF in the right insula (*t* = 5.47, Cohen’s *d* = 0.91), right parahippocampal gyrus (PHG; *t* = 5.37, Cohen’s *d* = 0.89), left cuneus (*t* = 4.76, Cohen’s *d* = 0.79), right inferior frontal gyrus (IFG; *t* = 4.46, Cohen’s *d* = 0.74), left caudate (*t* = 4.33, Cohen’s *d* = 0.72), right superior temporal gyrus (STG; *t* = 4.3, Cohen’s *d* = 0.71), right supramarginal gyrus (SMG; *t* = 4.09, Cohen’s *d* = 0.68), orbital part of inferior frontal gyrus (IOG; *t* = 3.91, Cohen’s *d* = 0.65), right superior frontal gyrus (SFG; *t* = 3.9, Cohen’s *d* = 0.65), and left cerebellar tonsil (*t* = 3.71, Cohen’s *d* = 0.61). Details see Table 2 and Figure 2. Notably, there were no regions showed significant decreased CVfALFF in FEP patients compared with HCs.

**TABLE 2:** Abnormal dfALFF in FES patients at baseline compared to healthy controls.

Brain region	MNI			Cluster size	T value	Cohen’s d
	X	Y	Z			
FES > HCs						
Right insula	42	12	−9	21	5.47	0.91
Right parahippocampal gyrus	33	−39	−9	12	5.37	0.89
Left cuneus	−3	−87	12	16	4.76	0.79
Right inferior frontal gyrus	63	12	21	12	4.46	0.74
Left caudate	−12	18	15	10	4.33	0.72
Right superior temporal gyrus	42	−18	−21	16	4.3	0.71
Right supramarginal gyrus	66	−36	27	10	4.09	0.68
Orbital part of inferior frontal gyrus	48	45	−9	12	3.91	0.65
Right superior frontal gyrus	0	39	39	12	3.9	0.65
Left cerebellar tonsil	−9	−48	−33	12	3.71	0.61
FES < HCs						
N/A	—	—	—	—	—	—

Abbreviations: dfALFF, dynamic fractional amplitude of low-frequency fluctuation; FES, first-episode schizophrenia; HCs, healthy controls.

**FIGURE 3:**
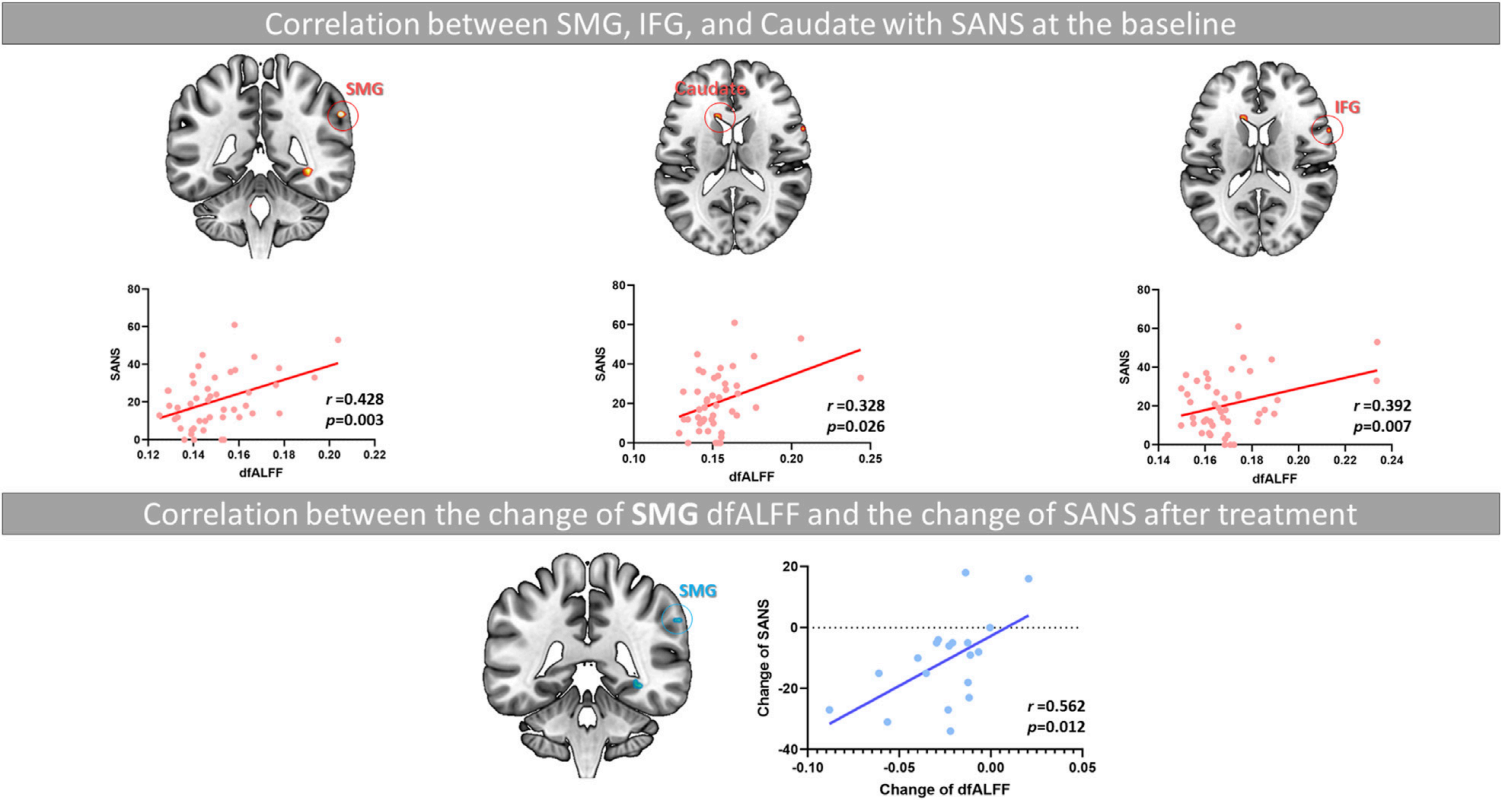
The relationship between the decrease in SMG instability and the improvement of negative symptoms in FES patients after long-term treatment. Note. SMG, supramarginal gyrus; IFG, inferior frontal gyrus; SANS, the Scale for the Assessment of Negative Symptoms; dfALFF, dynamic fractional Amplitude of Low Frequency Fluctuations.

As shown in Figure 3, in the correlation analysis at baseline, we observed the CVfALFF of the SMG, caudate, and IFG was positively correlated with SANS total scores (SMG, *r* = 0.428, *p* = 0.003; caudate, *r* = 0.328, *p* = 0.026; IFG, *r* = 0.392, *p* = 0.007).

### Effect of antipsychotic treatment on abnormal brain regions

As shown in Table 3 and Figure 2, after treatment, the abnormal increased CVfALFF at baseline in the SMG (*t* = −4.93, Cohen’s *d* = 0.82), PHG (*t* = −4.75, Cohen’s *d* = 0.79), caudate (*t* = −3.59, Cohen’s *d* = 0.59), IOG (*t* = −3.42, Cohen’s *d* = 0.57), insula (Cohen’s *d* = 0.57) and IFG (*t* = −2.97, Cohen’s *d* = 0.49) of the patients were significantly reduced.

**TABLE 3:** Effects of long-term atypical antipsychotic treatment on dfALFF in FES.

Brain region	MNI			Cluster size	T value	Cohen’s d
	X	Y	Z			
T2 < T1						
Right supramarginal gyrus	60	−45	36	6	−4.93	0.82
Right parahippocampal gyrus	33	−39	−9	5	−4.75	0.79
Left caudate	−15	21	15	5	−3.59	0.59
Orbital part of inferior frontal gyrus	48	54	−3	9	−3.42	0.57
Right insula	39	3	0	12	−3.42	0.57
Right inferior frontal gyrus	60	9	9	7	−2.97	0.49
T2 > T1						
N/A	—	—	—	—	—	—

Abbreviations: T1, time point at the baseline; T2, time point after treatment.

Due to the significant improvement in the SAPS and SANS total scores of most patients after treatment, we conducted a Pearson’s analysis to investigate the relationship between the reduction of CVfALFF in these brain regions after medication and the amelioration of clinical symptoms. As shown in Supplementary Table S4 and Figure 3, the results showed that the amplitude of changes in CVfALFF of the SMG (T2-T1) was positively correlated with the amplitude of changes in SANS (T2-T1) (*r* = 0.56, *p* = 0.012).

### Validation analysis

Our validation analysis using a window length of 30TRs also showed similar results (Supplementary Tables S5, S6).

## Discussion

To our knowledge, this study represents the first investigation using CVfALFF indicators to examine the impact of long-term antipsychotics on brain stability of regional activity in FES patients. We present two key findings. Firstly, at baseline, compared with HCs, FES patients exhibited increased CVfALFF across widespread regions that included the insula, PHG, cuneus, IFG, caudate, STG, SMG, IOG, SFG, and cerebellar tonsil. In these brain regions, we observed a positive correlation between CVfALFF in SMG, Caudate, IFG and the total score of SANS. Secondly, following long-term treatment, the heightened CVfALFF in the SMG, PHG, caudate, IOG, insula and IFG showed amelioration, and the amelioration in CVfALFF in the SMG after treatment was also associated with the resolution of negative symptoms.

In FES patients, heightened instability was observed in widespread brain regions, with similar findings reported in patients with schizophrenia and major depressive disorder (Braun et al., 2021; Yang et al., 2022; Wang et al., 2023). This indicated the potential brain activity instability that patients with severe mental disorders might possess. Several top-down approaches based on statistical dynamic frameworks have been used to study symptoms of schizophrenia, indicating that the generation of cognitive, negative, and positive symptoms of schizophrenia may be associated with the decreased signal-to-noise ratio and increased statistical fluctuations in different brain cortical networks, suggesting a close relationship between these symptoms and the instability of the neurodynamic system (Loh et al., 2007; Rolls, 2021). These studies suggests that due to reasons such as decreased NMDA receptor function, reduced neuronal spines, or decreased GABA neurotransmission, the firing state of cortical neurons becomes unstable or the firing rate decreases, leading to the instability of the neurodynamic system. This is consistent with the findings of our current study, where the severity of negative symptoms is associated with increased instability in three areas. Moreover, this variation is widely alleviated after long-term treatment, which suggests that long-term treatment may stabilize the firing state of cortical neurons in these cortical regions through relevant pharmacological mechanisms.

The SMG, located around the terminal of the ascending branch of the lateral sulcus, together with the angular gyrus, forms the inferior parietal lobule. The functions involved in this region include social cognition, working memory, and executive functions, among others (Palaniyappan and Liddle, 2012). In schizophrenia, the involvement of the inferior parietal lobe is associated with various impairments, including sensory integration, body image, self-concept, and executive function deficits (Torrey, 2007). In fMRI studies of patients with schizophrenia, chronic schizophrenia patients exhibit lower activity in the SMG during semantic priming (Jeong and Kubicki, 2010) and reduced activation of the SMG when facing negative semantic stimuli (Lee et al., 2019). A 6-week study of risperidone treatment showed that the SMG was one of the most prominent areas exhibiting reduced brain circuit function compared to FES patients at baseline (Nelson et al., 2020). Notably, the instability of SMG activity in this study was positively correlated with the severity of negative symptoms in patients, both at baseline and after long-term treatment. The relationship between the SMG and negative symptoms needs further exploration in the future.

The caudate is rich in DRD2 receptors and has been the focus of research on the dopaminergic system in schizophrenia for decades. In patients with schizophrenia, the caudate undergoes changes in morphology and function (Buchsbaum et al., 2003; Wada et al., 2012). Additionally, comprehensive genetic and transcriptional analysis of the caudate in schizophrenia has revealed new genetic associations and potential therapeutic targets related to dopamine metabolism (Benjamin et al., 2022). It is noteworthy that research has also shown increased activation of D1 receptors can ameliorate the instability of brain neural networks by enhancing the synaptic currents mediated by NMDA receptors (Loh et al., 2007), which may be related to the alleviation of the instability of caudate activity after long-term treatment in this study.

The insula plays a critical role in processing emotions and sensory stimuli (Wylie KP and Tregellas JR, 2010). In patients with schizophrenia, the functional connectivity between the insula and several key regions is impaired and this impaired functional connectivity may be a crucial factor in determining the severity of the disease (Tian et al., 2019; Sheffield et al., 2020). A 6-week antipsychotic treatment study on FES patients revealed a significant increase in glutamate levels in the insula following the treatment (Sonnenschein et al., 2022). Additionally, another study demonstrated improvements in abnormal dynamic functional connectivity patterns in the insula after an 8-week treatment with risperidone in FES patients (Duan et al., 2020b). In this study, it was found that the abnormal patterns of dynamic activity in the insula were also improved after long-term treatment in FES patients, highlighting the pivotal role of the insula in both short-term and long-term treatments.

The PHG is a crucial component of the limbic system, which has been found to be associated with cognitive impairments in individuals with schizophrenia (Schmitt and Falkai, 2023). Research has consistently shown that the PHG are linked to various cognitive functions, including processing speed, working memory, and language learning in patients with schizophrenia (Frith et al., 1991; Curtis et al., 2021; Guimond et al., 2016; van Erp et al., 2018). Research has suggested that antipsychotic medications can influence the PHG, potentially leading to improvements in memory function for patients (Tamminga et al., 2012). Long-term treatment with antipsychotic drugs has been shown to alleviate the high levels of activity instability seen in the PHG, highlighting the importance of this region in symptom amelioration for FES patients.

The IOG is a part of the default mode network (DMN). In the research of the neuropathology of schizophrenia, the DMN is considered one of the most relevant systems (Dong et al., 2018). Additionally, in patients with schizophrenia, the DMN is believed to be associated with both negative and positive symptoms (Whitfield-Gabrieli et al., 2009; Hare et al., 2019). It is worth noting that several studies on short-term antipsychotic medication treatment have found beneficial effects on DMN dysfunction (Zong et al., 2019; Duan et al., 2020a). Based on the results of this experiment, it also supplements the specific effects of long-term treatment on the DMN in FES patients. Similar to other brain regions, the IFG also undergoes changes following antipsychotic treatment, indicating the potential underlying mechanisms of IFG in long-term therapy.

However, there are several limitations that should be acknowledged in this study. Firstly, the follow-up patients were not sampled at a standardized time point, resulting in a non-uniform treatment duration for patients. This is due to the difficulty in obtaining follow-up data on patients receiving long-term antipsychotic treatment for an equivalent duration. Moreover, most clinical guidelines recommend a maintenance therapy duration of 6–24 months for relatively stable FES patients after acute treatment. Therefore, this study enrolled patients who received treatment for a duration within this range. It is advisable for future research to adopt a more detailed design that standardizes the treatment duration, as it would contribute to a more comprehensive exploration of the impact of long-term treatment on abnormal brain regions in FES patients. Secondly, the patients were derived from three different datasets, with the majority of HCs belonging to dataset #1. This disparity may introduce bias in the results pertaining to HCs. To address this concern, we utilized the scanning site as a covariate in order to mitigate any potential bias. Finally, our study lacked consistent monitoring of healthy controls over the same duration, potentially hindering our ability to mitigate the influence of time effects.

In conclusion, this study is the first to explore the impact of long-term medication on brain stability and successfully elucidates the widespread areas of activity instability in FES patients at baseline, as well as the improved stability in multiple brain regions following long-term treatment. Furthermore, this study demonstrates the association between abnormal dfALFF in the SMG and negative symptoms. These findings provide new insights into the mechanisms underlying the long-term treatment of FES patients.

## Data Availability

The raw data supporting the conclusion of this article will be made available by the authors, without undue reservation.
