# Supplementary material for: Effects of long-term antipsychotic medication on brain instability in first-episode schizophrenia patients: a resting-state fMRI study
Source: Front Pharmacol. 2024 May 23;15:1387123. doi: 10.3389/fphar.2024.1387123 (PMC11153814; doi:10.3389/fphar.2024.1387123)
Supplement: Supplementary file 1 [file DataSheet1.docx]

**Supplementary Material**

**Methods**

***S1*** *Criteria for patient acceptance and exclusion*

The acceptance criteria for FES patients: (1) Age range from 14 to 40 years old; (2) (2) Han Chinese ethnicity; (3) 6 years of education or above; (4) right-handed preference; (5) meeting DSM-IV criteria for schizophrenia; (6) first episode (illness duration < 18 months), having antipsychotics for less than 2 weeks. The exclusion criteria for FES patients: (1) Mental disorders caused by drugs or psychoactive substances; (2) Organic mental disorders; (3) contra-indications to MRI scanning; (4) history of unconsciousness lasting longer than 5 minutes; (5) patients who received electroconvulsive therapy (ECT) and transcranial magnetic stimulation (TMS) treatments.

***S2*** *details of antipsychotics drugs*

The following antipsychotic drugs were used in 22 follow-up patients: risperidone (12 patients), ziprasidone (3 patients), olanzapine (2 patients), aripiprazole (1 patients), amisulpride (1 patients), and combined atypical antipsychotics (3 patients).

***S3*** *Image data acquisition and preprocessing*Resting-state functional magnetic resonance imaging (fMRI) images of Dataset 1#、and Dataset 3# obtained using Philips Gyroscan Achieva 3.0T scanner in the axial direction. And Dataset 2# was collected using Siemens Achieva 3.0T scanner. Their gradient-echo echo-planar imaging sequences were used with the following parameters respectively: (1) Dataset 1#: TR = 2000ms, TE = 30ms, slice thickness = 4 mm, slice gap = 4 mm, acquisition matrix = 64×64, field of view = 240×240 mm^2^, flip angle = 90°, and voxel size = 1.67×1.67×4 mm^3^. Each brain volume comprised 36 slices, and the functional run contained 250 image volumes. (2) Dataset 2#: TR = 2000ms, TE = 30ms, slice thickness = 4 mm, slice gap = 5 mm, acquisition matrix = 64×64, field of view = 250×250 mm^2^, flip angle = 80°, and voxel size = 4×4×5 mm^3^. Each brain volume comprised 32 slices, and the functional run contained 216 image volumes. (3) Datasets 3#: TR = 2000ms, TE = 30ms, slice thickness = 4 mm, slice gap = 4 mm, acquisition matrix = 72×68, field of view = 230×230 mm^2^, flip angle = 90°, and voxel size = 1.6×1.6×4 mm^3^. Each brain volume comprised 32 slices, and the functional run contained 250 image volumes.

The volume of the initial 10 time points was discarded for the stability of the scanner and the adaptation of the participants to the environment. The remaining volumes were corrected for the acquisition delay between slices, followed by a realignment to the middle volume. To ensure data quality, participants were excluded if their head motion were more than 3.0 mm or 3.0° during resting-state fMRI. After realignment, images were then spatially normalized into Montreal Neurological Institute standardized space resampled to 3×3×3 mm^3^ voxels and spatially smoothed with a Gaussian kernel (full width at half-maximum = 8 mm). Linear detrending was conducted and followed by nuisance covariates regression including the signals from cerebrospinal fluid and white matter, 6-parameter rigid-body motion correction parameters as well as the global mean signal. We also performed the head motion scrubbing regression to eliminate the confounding effect of subtle head movement. Finally, signal from each ‘bad’ frame defined as frame-wise displacement (FD) > 0.5 and its neighbors (1 frame before and 2 frames after) were flagged for regression. Subsequently, all time-series was then high- and low-pass filtered at 0.01 Hz and 0.1 Hz, respectively.

**Table**

***S1*** *Composition details of 98 participants from 3 datasets*

| **clinical centers** | **dataset** | **Compositions of participants** | | |  |
| --- | --- | --- | --- | --- | --- |
|  |  | **FES at baseline** | **HC** | **FES at follow-up** |  |
| The Second Xiangya Hospital | 1# | 19 | 53 | 16 |  |
|  | 2# | 8 | 0 | 4 |  |
| Queen Mary Hospital, The University of Hong Kong | 3# | 25 | 0 | 4 |  |
|  |  |  |  |  |  |
| *Note: FES, first-episode schizophrenia patients; HC, healthy controls.* | | | | |  |

***S2*** *Clinical symptoms comparison between dropout and follow-up patients at baseline*

| **Clinical symptoms** | **Patients** | | **t** | ***p*** |  |
| --- | --- | --- | --- | --- | --- |
|  | **Dropout** | **Follow-up** |  |  |  |
| SAPS | 29.84±14.69 | 23.19±14.42 | -1.58 | >0.05 |  |
| SANS | 23.42±16.52 | 21.09±16.12 | -0.49 | >0.05 |  |
| *Note: SANS, scale for the assessment of negative symptoms; SAPS, scale for the assessment* | | | | |  |
|  |  |  |  |  |  |

***S3*** *Reduction of SANS total score and SAPS total score in follow-up patients after long-term treatment*

| **Clinical symptoms** | **Mean±SD (%)** | **Frequency** |  |
| --- | --- | --- | --- |
|  |  | **(reduction rate≥30%)** |  |
| SAPS | 80.18±28.81 | 20 |  |
| SANS | 18.36±118.28 | 17 |  |
| *Note: SANS, scale for the assessment of negative symptoms; SAPS, scale for the assessment* | | |  |
|  |  |  |  |

**S4** *Factors related to different brain regions after long-term treatment*

| **brain region (T2-T1)** | **Change in SANS Total Score (T2-T1)** | |  | **Change in SAPS Total Score (T2-T1)** | |  | **chlorpromazine equivalence** | |  |
| --- | --- | --- | --- | --- | --- | --- | --- | --- | --- |
|  | ***r*** | ***p*** |  | ***r*** | ***p*** |  | ***r*** | ***p*** |  |
| **SMG** | **0.562** | **0.012** |  | -0.191 | 0.42 |  | -0.031 | 0.892 |  |
| **PHG** | 0.127 | 0.604 |  | -0.292 | 0.212 |  | -0.026 | 0.359 |  |
| **Caudate** | 0.243 | 0.317 |  | -0.032 | 0.893 |  | 0.055 | 0.809 |  |
| **IOG** | 0.295 | 0.22 |  | -0.276 | 0.238 |  | 0.079 | 0.728 |  |
| **Insula** | 0.223 | 0.359 |  | -0.149 | 0.531 |  | -0.114 | 0.613 |  |
| **IFG** | 0.41 | 0.081 |  | 0.059 | 0.805 |  | 0.051 | 0.823 |  |
| T1, time point at the baseline; T2, time point after treatment; SMG, Supramarginal Gyrus; PHG, parahippocampal gyrus; IOG, Orbital Part of Inferior Frontal Gyrus; IFG, Inferior Frontal Gyrus. SANS, scale for the assessment of negative symptoms; SAPS, scale for the assessment | | | | | | | | |  |
|  |  |  |  |  |  |  |  |  |  |

**S5** *Abnormal dfALFF in FES patients at baseline compared to healthy controls. (validation analysis using a window length of 30TRs)*

|  | | | | | |
| --- | --- | --- | --- | --- | --- |
| **brain region** |  | **MNI** |  | **T Value** | **Voxels** |
|  | **X** | **Y** | **Z** |  |  |
| **FES > HCs** |  |  |  |  |  |
| Left Cuneus | -3 | -96 | 6 | 4.81 | 15 |
| Right Parahippocampal Gyrus | 33 | -39 | -9 | 4.77 | 12 |
| Right Insula | 42 | 0 | -3 | 4.37 | 12 |
| Left Caudate | -9 | 12 | 21 | 4.25 | 10 |
| Right Superior Temporal Gyrus | 39 | -15 | -24 | 4.24 | 13 |
| Right Inferior Frontal Gyrus | 63 | 15 | 21 | 3.92 | 12 |
| Right Superior Frontal gyrus | 0 | 39 | 39 | 3.88 | 7 |
| Orbital Part of Inferior Frontal Gyrus | 48 | 45 | -9 | 3.87 | 11 |
| Right Supramarginal Gyrus | 63 | -42 | 36 | 3.74 | 10 |
| Left Cerebellar Tonsil | -9 | -48 | -33 | 3.4 | 12 |
| Right Limbic Lobe | 9 | 3 | 42 | 3.19 | 10 |
| **FES < HCs** |  |  |  |  |  |
| N/A | - | - | - | - | - |
| *Abbreviations: dfALFF, Dynamic Fractional Amplitude of Low-Frequency Fluctuation; FES, First-Episode Schizophrenia; HCs, healthy controls.* | | | | | |

**S6** *Effects of long-term atypical antipsychotic treatment on dfALFF in FES. (validation analysis using a window length of 30TRs)*

| **brain region** |  | **MNI** |  | **T Value** | **Voxels** |
| --- | --- | --- | --- | --- | --- |
|  | **X** | **Y** | **Z** |  |  |
| **T2<T1** |  |  |  |  |  |
| Right Supramarginal Gyrus | 60 | -45 | 36 | -4.25 | 6 |
| Right Parahippocampal Gyrus | 33 | -39 | -9 | -4.17 | 5 |
| Orbital Part of Inferior Frontal Gyrus | 48 | 48 | 0 | -3.99 | 5 |
| Left Cuneus | -3 | -96 | 6 | -3.15 | 6 |
| Right Insula | 45 | -3 | 0 | -3.03 | 9 |
| **T2>T1** |  |  |  |  |  |
| N/A | - | - | - | - | - |
| Abbreviations: T1, time point at the baseline; T2, time point after treatment. | | |  |  |  |
